# Warburg and Crabtree Effects in Premalignant Barrett's Esophagus Cell Lines with Active Mitochondria

**DOI:** 10.1371/journal.pone.0056884

**Published:** 2013-02-27

**Authors:** Martin T. Suchorolski, Thomas G. Paulson, Carissa A. Sanchez, David Hockenbery, Brian J. Reid

**Affiliations:** 1 Molecular and Cellular Biology Department, University of Washington, Seattle, Washington, United States of America; 2 Human Biology Division, Fred Hutchinson Cancer Research Center, Seattle, Washington, United States of America; 3 Public Health Sciences Division, Fred Hutchinson Cancer Research Center, Seattle, Washington, United States of America; 4 Clinical Research Division, Fred Hutchinson Cancer Research Center, Seattle, Washington, United States of America; 5 Department of Medicine, University of Washington, Seattle, Washington, United States of America; 6 Department of Genome Sciences, University of Washington, Seattle, Washington, United States of America; University of Texas Health Science Center at San Antonio, United States of America

## Abstract

**Background:**

Increased glycolysis is a hallmark of cancer metabolism, yet relatively little is known about this phenotype at premalignant stages of progression. Periodic ischemia occurs in the premalignant condition Barrett's esophagus (BE) due to tissue damage from chronic acid-bile reflux and may select for early adaptations to hypoxia, including upregulation of glycolysis.

**Methodology/Principal Findings:**

We compared rates of glycolysis and oxidative phosphorylation in four cell lines derived from patients with BE (CP-A, CP-B, CP-C and CP-D) in response to metabolic inhibitors and changes in glucose concentration. We report that cell lines derived from patients with more advanced genetically unstable BE have up to two-fold higher glycolysis compared to a cell line derived from a patient with early genetically stable BE; however, all cell lines preserve active mitochondria. In response to the glycolytic inhibitor 2-deoxyglucose, the most glycolytic cell lines (CP-C and CP-D) had the greatest suppression of extra-cellular acidification, but were able to compensate with upregulation of oxidative phosphorylation. In addition, these cell lines showed the lowest compensatory increases in glycolysis in response to mitochondrial uncoupling by 2,4-dinitrophenol. Finally, these cell lines also upregulated their oxidative phosphorylation in response to glucose via the Crabtree effect, and demonstrate a greater range of modulation of oxygen consumption.

**Conclusions/Significance:**

Our findings suggest that cells from premalignant Barrett's esophagus tissue may adapt to an ever-changing selective microenvironment through changes in energy metabolic pathways typically associated with cancer cells.

## Introduction

Two well-known differences in energy metabolism have been shown to exist between normal and cancer cells: the Warburg and Crabtree effects. In 1927, Otto Warburg discovered that cancer cells produce twice as much ATP as normal cells through glycolysis, even in oxygenated tumors [1]. Warburg also reported reduced oxygen consumption in several cancers and postulated that damaged mitochondria were the cause of increased glycolysis [2]. This hypothesis was later contested as non-glycolytic cancers were discovered [3] and it was found that, even in primarily glycolytic tumors, mitochondria were essential for proliferation, metastasis, and were even hyperactive in some tumors [4], [5], [6]. Although mitochondria have been traditionally studied as sources of cellular energy, these organelles also provide vital biosynthetic, anabolic and apoptotic functions, some of which are altered in cancer cells [7]. Cancer cells have also been shown to be different from normal cells by reversibly down-regulating their oxygen consumption in response to increases in glucose: the Crabtree effect [8]. Both of these metabolic effects are thought to be protective and contribute to cancer cell survival in a dynamic environment periodically experiencing hypoxia [9]. Given that hypoxia and glycolytic adaptation is common in a large variety of cancers, these metabolic adaptations have been targeted for therapy; however clones refractory to treatment may evolve from the clonal heterogeneity in cancers [10]. Although the Warburg and Crabtree effects have been well characterized in cancer cells for nearly a century, their role in premalignant conditions, which are more successfully treated, have not been closely investigated.

Barrett's esophagus (BE) is a premalignant condition in which intestinal metaplasia replaces normal esophageal squamous epithelium and is associated with an increased risk of esophageal adenocarcinoma (EA) [11]. BE is a unique model for investigating neoplastic progression *in vivo* because periodic endoscopic surveillance is recommended in BE patients for early detection of cancer, allowing intermediate stages of intestinal metaplasia to be characterized and monitored at regular intervals [12], [13]. While a panel of biomarkers, including 9pLOH, 17pLOH, tetraploidy, and aneuploidy provide a method for predicting BE patient progression to cancer (RR = 38.7; 95% CI 10.8–138.5, p<0.001) [14], little is known about the state of energy metabolism in BE. hTert-immortalized BE cell lines containing similar genomic alterations to primary biopsies *in vivo* have been derived from primary premalignant tissue at early and late stages of progression and provide an opportunity to study cellular metabolism of premalignant lesions *in vitro* [15].

BE develops as an adaptation to the erosive environment of acid-reflux with periodic hypoxic events due to ulceration and ischemia [16]. Given that esophageal adenocarcinoma (EA) has been shown to display the Warburg effect [17], premalignant BE cells may be subject to selective pressure to adapt to variable oxygen and glucose concentrations before progression to EA. Although BE rarely displays levels of glucose flux that can be detected by positron emission tomography (PET) [18], Georgakoudi et al have successfully discriminated localized regions of BE from normal tissue by fluorescent tissue imaging of regions with elevated NADH/NADPH levels, characteristic of hypoxic tissue [19]. Several markers of hypoxia, including HIF-2α and VEGF, have also been reported previously in advanced BE [20], [21], however levels of glycolysis and oxidative phosphorylation have never been directly characterized.

Since metaplastic esophageal mucosa from individuals with BE have phenotypes suggesting selective adaptation to hypoxia, we hypothesized that later-stage BE cell lines display increased glycolysis and decreased oxidative phosphorylation, compared to early-stage cell lines. Also, since glucose levels would fluctuate with periodic tissue damage in BE we hypothesized that later-stage BE cell lines display the Crabtree effect. To test our hypotheses, we compared metabolic rates of glycolysis and oxidative phosphorylation in four cell lines derived from individuals with BE at different stages of progression and measured changes in response to metabolic inhibitors and varying glucose concentrations. If BE cell lines representing different stages of progression to cancer have distinct metabolic features, then it may be possible to develop novel metabolic biomarkers for EA risk or therapeutic targets for pre-malignant stages.

## Materials and Methods

### Ethics Statement

Cell lines and control samples were derived from participants who were enrolled in the Seattle Barrett's Esophagus Study, which was approved by the Human Subjects Division of the University of Washington in 1983 and renewed annually thereafter with reciprocity from the Fred Hutchinson Cancer Research Center Institutional Review Board (FHCRC IRB) from 1993 to 2001. Since 2001, the study has been approved by the FHCRC IRB with reciprocity from the University of Washington Human Subjects Division.

### Tissue Culture

hTert-immortalized Barrett's esophageal cell lines CP-A, CP-B, CP-C and CP-D were previously derived from premalignant Barrett's esophagus tissue [15] and adapted for serum-free growth by the Carlo Maley lab (University of California San Francisco). Research participants from whom the biopsies for cell lines were derived were followed in a separate longitudinal study that evaluated changes in genetic biomarkers and progression to cancer. CP-A was derived from a patient with metaplastic BE at early-stages with wild-type TP53 and low levels of genetic instability, while CP-B, CP-C and CP-D were derived from patients with later-stage BE containing mutations and/or deletions in both copies of TP53 and high levels of genetic alterations (Text S1 and Table S1). CP-B was derived from a patient that was also diagnosed with EA, but from a biopsy taken at a different level in the esophagus. Serum-dependent cell line genetic alterations were previously verified to be similar to those found in patient biopsies [15]. The cell lines were cultured in keratinocyte serum-free media (KSFM) with 5 µg/L human epithelial growth factor and 50 mg/L bovine pituitary extract (media and supplements from Invitrogen).

Since Barrett's epithelium is a metaplasia which is of different origin than normal esophageal squamous epithelium, there is no ‘normal’ tissue control; therefore CRL-4001/BJ-5ta (Geron Corporation, ATCC), a genetically stable immortalized foreskin fibroblast cell line, was cultured as a control cell line in four parts of DMEM Dulbecco's Modified Eagle's Medium (Invitrogen), containing 4 mM L-glutamine and 4.5 g/L glucose, to one part of Medium 199 (Sigma), supplemented with 0.01 mg/ml hygromycin B (all supplements from Sigma) and 10% fetal bovine serum (Gemini Bioproducts). OE-33, an esophageal adenocarcinoma line (Health Protection Agency, UK) [22], was cultured as a control cancer cell line in RPMI 1640 (Invitrogen), supplemented with 2 mM Glutamine (Invitrogen) and 10% fetal bovine serum (Gemini Bioproducts). Since OE-33 adhered poorly to Seahorse assay plates, assays with this cell line could not be normalized. We also investigated two cell lines derived from esophageal squamous epithelium as potential controls, but the EPC-2 cell line (from the Carlo Maley lab, UCSF) was aneuploid (data not shown) and HET-1A cell-line was transformed by SV40 T-antigen, making them unsuitable as ‘normal’ esophageal squamous controls (Text S1).

All cell lines were grown in a humidified 37°C incubator with 5% CO_2_, and passaged upon reaching 50–80% confluency, once to twice weekly. To minimize over-trypsinization, passaging was performed using 0.05% Trypsin-EDTA (Invitrogen).

Several esophageal adenocarcinoma cell lines in existing cell banks have been reported to be contaminated and/or misidentified [23]. We therefore confirmed that the cell line DNA matched the normal DNA from each of the individuals from whom the BE cell lines were originally derived, using the AmpFlSTR Identifiler kit (Applied Biosystems). In addition, we evaluated the cell lines by DNA content flow cytometry (data not shown). Cell cultures were confirmed to be free of mycoplasma by MycoSensor PCR kit (Stratagene). Cell lines were refreshed from stock after 20 passages, or upon detection of changes in DNA content.

### Seahorse XF24 metabolic measurements

The Seahorse XF24 flux analyzer (Seahorse Bioscience Inc, North Billerica, MA, USA) is capable of simultaneously measuring changes in pH and oxygen levels from cells seeded on 24-well plates. Extracellular acidification rates (ECAR), representative of glycolysis, and oxygen consumption rates (OCR), representative of oxidative phosphorylation, were measured on the Seahorse XF24. While carbon dioxide produced by cells also acidifies the culture medium, glycolysis accounts for approximately 80% of the ECAR, and therefore ECAR has been shown to be consistent with the rate of lactate production by cells [24], [25].

Two days prior to seeding Seahorse plates, cell lines were passaged and seeded at cell densities that produce 50–70% confluency and consistent rates of proliferation. Prior to harvesting and re-seeding, the cell confluency was visually inspected.

Since ECAR and OCR measurements with the Seahorse instrument are confined to the media beneath the fluorescent sensors, localized differences in cell density contribute to noise in the Seahorse assay. Thus, consistent cell seeding is critical to minimize measurement noise. Optimal cell densities were established for Seahorse plates by plating two seeding densities for each cell line and determining well-to-well variability in ECAR and OCR. Since ECAR and OCR were highly reproducible for each seeding density, to avoid the potential for over-growth, the lower of the two seeding densities was determined to be optimal. To plate cells for Seahorse measurement, cells were concentrated in 100 µl medium specific to the cell line (described in Tissue Culture methods) and gently seeded with a pipette tip positioned at a 45-degree angle into 24-well Seahorse culture plates (Seahorse Bioscience Inc.). Three hours later, cells had adhered to the wells and 150 µl of medium was added. After overnight growth, medium was replaced with buffer-free Seahorse assay medium: Dulbecco's Modified Eagle's Medium (Sigma) supplemented with 5 mM glucose, 2 mM glutamine (Invitrogen), 10 mg/L phenol red and 1.85 g/L NaCl (all supplements from Sigma, except glutamine). The plates were equilibrated for one hour in a 37°C room-air incubator and sensors were calibrated in calibration solution (Seahorse Bioscience Inc). The plates were then evaluated on the Seahorse XF24. Five consecutive measurements were obtained on each well; however since the first OCR measurement consistently varied by more than two standard deviations from the subsequent measurements, it was discarded. Outlier data from wells that produced two-fold or greater increases in ECAR or OCR standard deviation when pooled were excluded from the analysis, since noisy sensors or poor signal from uneven seeding were suspected. Data from three to four wells per experiment were combined across at least six experiments for each cell line.

Preliminary analysis was performed using the Seahorse XF24 software (Version 1.7.0.74). Statistical analyses of differences in ECAR and OCR between cell lines were performed using ANOVA and Tukey-Kramer test on STATA software (Version 11.1, http://www.stata.com).

### Response to metabolic inhibitors measurements

Glycolytic inhibition was tested with 2-deoxyglucose (2-DG), a glucose analogue that competitively inhibits hexokinase activity in the first enzymatic step of glycolysis. Changes in ECAR and OCR were measured on the Seahorse XF24 in response to addition of 2-DG in consecutive injections that produced final concentrations of 1, 10, 25 and 50 mM. Five consecutive measurements were performed per well prior to and after each addition. Differences between cell line changes in ECAR and OCR at 50 mM 2-DG were analyzed with ANOVA and Tukey-Kramer tests using STATA software.

2,4-dinitrophenol (2,4-DNP), a well-established protonophore and mitochondrial uncoupling agent, causes depolarization of the inner mitochondrial membrane, resulting in a dramatic arrest of ATP generation. Cell line changes in ECAR and OCR were measured on the Seahorse XF24, in response to addition of 2,4-DNP in consecutive injections that produced final concentrations of 2, 20, 50 and 200 µM. Five consecutive measurements were performed per well, prior to and after each addition. In most cell lines, 200 µM 2,4-DNP produced a sudden collapse of OCR below baseline levels, indicating toxicity, thus only data from 2 to 50 µM 2,4-DNP were used for further analysis. Differences between cell line changes in ECAR and OCR at 50 µM 2,4-DNP were analyzed with ANOVA and Tukey-Kramer tests using STATA software.

Oligomycin, an ATP synthase inhibitor, was used to measure the amount of OCR which is dependent on ATP synthesis in each cell line. Cell line changes in ECAR and OCR were measured on the Seahorse XF24, in response to addition of 3.2 µM oligomycin. Five consecutive measurements were performed per well, prior to and after addition of the inhibitor. By subtracting the residual OCR following inhibitor addition from the total OCR prior to treatment, the OCR dependent on oxidative phosphorylation was obtained.

### Crabtree effect measurements

Cell lines were cultured in Seahorse plates as described above, except glucose-free buffer-free assay medium was substituted for the standard Seahorse assay medium. Following room air and buffer-free media equilibration and sensor calibration, the plates were run on the Seahorse XF24 instrument. Cell line changes in ECAR and OCR were measured in response to addition of glucose in consecutive injections that produced final concentrations of 0.25, 0.5 and 5 mM. CCCP (an uncoupling agent similar to 2,4-DNP) was then added at 0.5 µM final concentration to verify that mitochondria remained functional despite a drop in OCR following glucose addition. Although CCCP has a lower dynamic range than 2,4-DNP, its purpose in this experiment was to fully uncouple the mitochondria and not titrate. Five consecutive measurements were obtained prior to and following each addition. Data were evaluated for inter-replicate variability as above and pooled from four experiments. Differences between cell line changes in ECAR and OCR at 5 mM glucose were analyzed with ANOVA and Tukey-Kramer tests using STATA software.

### Seahorse data normalization per cell by image cytometric cell counting

Following each experiment, Seahorse assay media was replaced with 50 µl freezing media (Minimal Essential Media (Gibco) with 10% DMSO (Sigma), 5% heat deactivated fetal calf serum, 5 mM Hepes Buffer) and stored at −25°C. On the week of cell counting, plates were thawed, washed with PBS at room temperature, fixed with 70% ethanol for 10 minutes at 4°C, washed again with PBS and stained with 5 µg/ml DAPI. The plates were stored at 4°C in the dark until data collection. Image data were acquired using a Nikon-Ti microscopy platform equipped with a Sutter Instruments Lambda LS 300 W xenon arc lamp, 360/BP40 excitation and 460/BP50 emission filters, and automated stage. Image cytometry analysis was performed using ImageJ (Version 1.45i, http://rsbweb.nih.gov/ij/index.html) with a software macro that identified and counted nuclei. In each well, cells were imaged in four separate fields of view, each located in a distinct quadrant and visually verified to contain no staining artifacts. The mean from the four fields was extrapolated to the area of the well to estimate total number of cells. ECAR and OCR values were normalized using these cell counts within the Seahorse XF24 software (Version 1.7.0.74).

### Nuclear genome characterization

BE cell lines were analyzed for somatic genetic alterations (copy number alterations or copy-neutral LOH) using Illumina Omni-quad 1 M SNP arrays. DNA was isolated from an early passage of each cell line as well as from constitutive normal tissue (blood or gastric tissue) from the individual from which each cell line originated. Samples were processed and hybridized according to manufacturer's protocol. Data were initially processed using Illumina Bead Studio, and regions of gain, loss and copy neutral LOH were determined using Partek Genomics Suite v6.5.

EPC-2, CRL-4001 or OE-33 were excluded from nuclear genome characterization since they did not have patient-matched normal controls.

### Mitochondrial genome characterization

Cell line mitochondrial genomes were assessed using MitoChip v2.0 oligonucleotide arrays from Affymetrix. Sequences were compared to matched constitutive normal samples to ensure the alterations detected were somatic changes. Since EPC-2, CRL-4001 or OE-33 did not have patient-matched normal controls, they were excluded from mitochondrial genome characterization. For cell lines, DNA was extracted by standard protocol on Qiagen columns (Germantown, MD, USA). For constitutive DNA, DNA was extracted from gastric biopsies using Qiagen columns or Gentra DNA isolation kit (Minneapolis, MN, USA). mtDNA was amplified using two sets of PCR primers and samples were quantitated, fragmented and labeled according to the MitoChip v2.0 protocol.

MitoChip data were then analyzed using Affymetrix GCOS v1.3d and GSEQ v4.1 software and the putative mutations were verified on both forward and reverse strands. Cell line alterations that were detected were compared with matched constitutive DNA. Further characterization of the location of mutations, affected mitochondrial genes, and mitochondrial haplogroup for each cell line were determined using the Mitomaster mtDNA sequence analysis program [26].

### Mitochondrial mass measurements by flow cytometry

All cell lines were grown to 50–70% confluency and harvested with 0.05% trypsin. Cells were centrifuged at 100 g for six minutes and resuspended in KSFM media (Invitrogen) to a concentration of 4 million cells/ml.

To assess mitochondrial mass, cells were then diluted 1∶2 and stained for 30 minutes at 37°C in a staining solution containing final concentrations of 10 nM mitotracker green, 10 µM Hoechst 33342 and 5 µM Sytox Orange (all dyes from Invitrogen). Samples were measured by flow cytometry on the Beckton-Dickinson LSR-2 instrument. Dead cells were gated out using Sytox Orange, and were less than 0.1% for each cell line. Analysis was performed using FlowJo software (Treestar, Ashland, OR) on whole-cell and G1-phase fractions that were gated using the Hoechst 33342 cell cycle profile.

## Results

### Late-stage Barrett's esophagus cell lines display higher ECAR in glycolysis and oxidative phosphorylation

To determine if late-stage BE cell lines demonstrate increased glycolysis and/or decreased oxidative phosphorylation compared to an early-stage BE cell line, extracellular acidification rates (ECAR) and oxygen consumption rates (OCR) were measured using the Seahorse XF24 analyzer in an early-stage BE cell line (CP-A), three late-stage BE cell lines (CP-B, -C, and -D), and a fibroblast control cell line (CRL-4001). Difficulties associated with cell adhesion prevented normalization of the EA cell line OE-33 and comparison of absolute OCR and ECAR to the BE cell lines, however relative results were still reported (Text S1).

**Figure 1:**
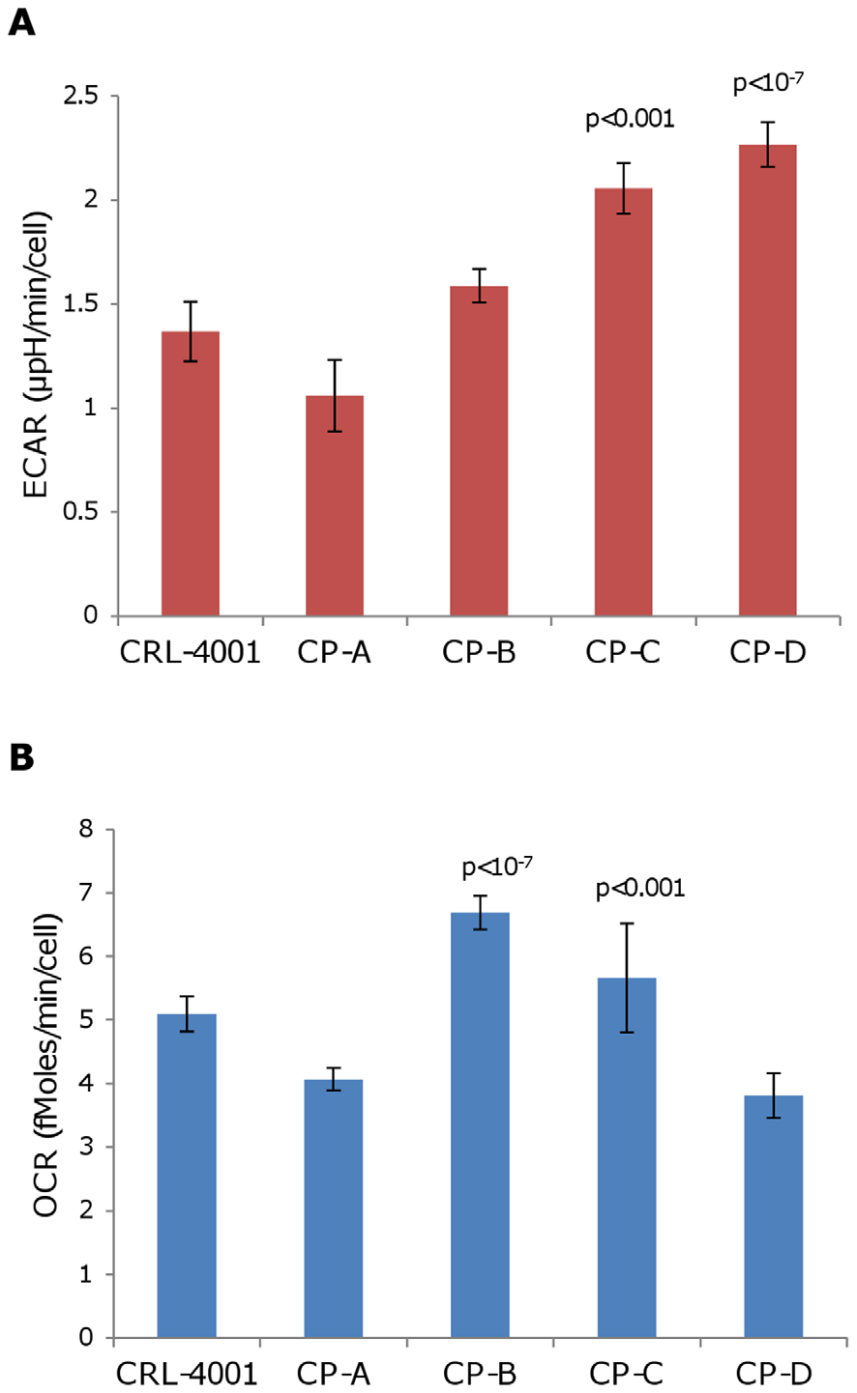
CP-D demonstrates a significantly elevated ECAR than other BE cell lines. Shown above are mean measures of total (a) ECAR/cell and (b) OCR/cell. Error bars represent standard-deviation of mean between experiments (N = 6–11). Each experiment consisted of 3 to 4 replicate wells per cell line with four serial measures performed on each well. p-values (Tukey-Kramer test) of statistically significant differences from CP-A are shown.

ECAR in CP-D was two-fold higher than in CP-A (p<10^−7^; Figure 1a and Table S2). ECAR in CP-B and CP-C were also higher than in CP-A, but only reached significance in CP-C (p<0.001). OCR was higher in CP-B and CP-C than in CP-A (p<10^−7^ and p<0.001 respectively; Figure 1b and Table S2). OCR in CP-D was not found to be statistically different than in CP-A. There was no correlation found between levels of ECAR and OCR for the cell lines (R = 0.288).

To confirm that OCR is dependent on ATP synthesis in each cell line, ECAR and OCR changes were also measured in response to treatment with the ATPase inhibitor oligomycin. All cell lines demonstrated a 57–73% decrease in OCR, which corresponds to their OCR that is dependent on ATP synthesis by oxidative phosphorylation (Table S4). In response to oligomycin treatment, CP-D demonstrated a lower increase in ECAR than the other BE cell lines (Table S4).

### CP-D is the most sensitive cell line to glycolytic inhibition by 2-deoxyglucose, but compensates with mitochondrial activity

To test the effects of glycolytic inhibition on the BE cell lines, ECAR and OCR changes were measured in response to 2-deoxyglucose (2-DG), a competitive inhibitor of the glycolytic pathway. If a cell line has elevated glycolysis (such as CP-C and CP-D), a greater decrease in ECAR would be expected in response to 2-DG compared to cells that are less glycolytic. In cell lines that have functional mitochondria, OCR would also increase to compensate for the decrease in glycolytic ATP production. If OCR does not increase in response to glycolytic inhibition, then the cells would have adequate ATP production by oxidative phosphorylation alone, have dysfunctional mitochondria or are unable to oxidize alternative substrates (eg. glutamine or fatty acids). If OCR instead decreases in response to glycolytic inhibition, then the rate of oxidative phosphorylation in that cell line would likely be limited by the rate of synthesis of pyruvate or other mitochondrial substrates.

**Figure 2:**
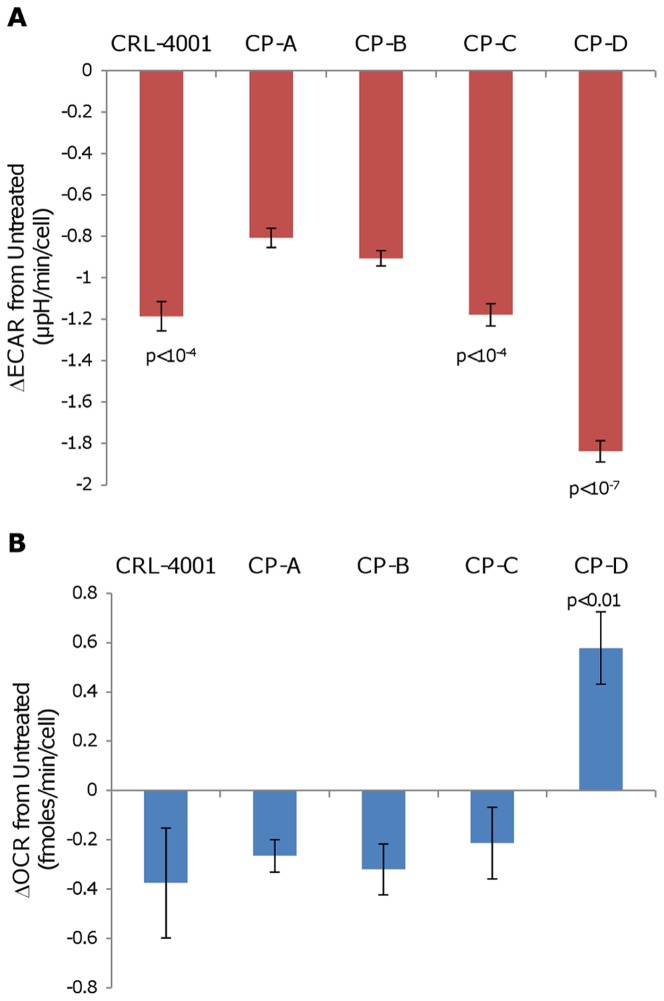
CP-D displays a greater OCR and ECAR response to glycolytic inhibition than other cell lines. Following addition of 50 mM 2-DG, total (a) ECAR and (b) OCR were measured on the Seahorse XF24 analyzer for each cell line and changes versus untreated baseline are plotted. Error bars represent standard-deviation of means between experiments (N = 2–4). Each experiment consisted of 3–4 replicate wells per cell line with four serial measures performed on each well. p-values (Tukey-Kramer test) of statistically significant differences from CP-A are shown.

In response to 2-DG, ECAR decreased more in CP-C and CP-D than in CP-A (p<10^−4^ and p<10^−7^ respectively; Figure 2a and Table S3). In response to 2-DG, OCR in CP-A, CP-B and CP-C decreased, while OCR in CP-D increased (p<0.01; Figure 2b and Table S3).

### CP-C and CP-D are the least sensitive BE cell lines to mitochondrial uncoupling by 2,4-dinitrophenol

Uncoupling agents such as 2,4-dinitrophenol (2,4-DNP) dissipate mitochondrial potential and arrest ATP generation. In cases where mitochondrial potential exceeds the reversal potential of the adenine nucleotide translocase (ANT), mitochondrial ATPase would consume ATP to pump protons across the inner mitochondrial membrane to restore potential [27]. As a result of these two events, uncoupled cells would rapidly deplete their ATP levels and upregulate glycolysis to compensate. Since glycolytic cell lines are known to have greater resistance to uncoupling agents and mitochondrial poisons [28], [29], we tested cell line ECAR and OCR responses to 2,4-DNP. If the cells are more glycolytic, a lower increase in ECAR would result in response to uncoupling, due to 1) lower disturbance in ATP/ADP levels since the cells are less reliant on oxidative phosphorylation and/or 2) an inability to further increase glycolysis beyond its already elevated levels. In cells having uncoupled mitochondria or a lower ADP/ATP ratio, a lower OCR increase would result upon 2,4-DNP addition, since the mitochondria are already functioning near maximum respiratory capacity. The maximum increase in OCR in uncoupled mitochondria compared to untreated is also defined as ‘mitochondrial reserve capacity’ and reflects the dynamic potential of the mitochondria to adapt to different energetic requirements of the cell.

**Figure 3:**
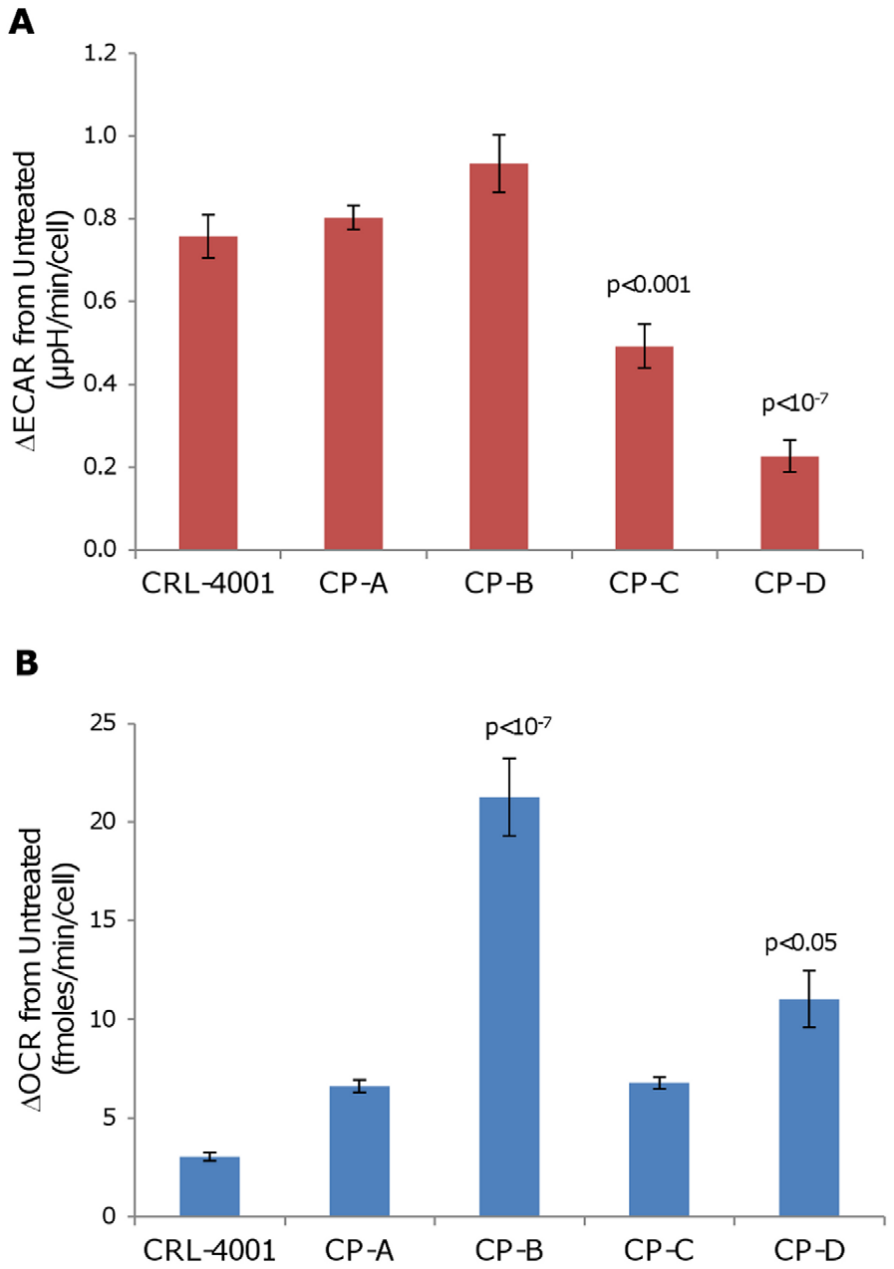
CP-C and CP-D display a lower ECAR increase after mitochondrial uncoupling than other cell lines. Following addition of 50 µM 2,4-DNP, total (a) ECAR and (b) OCR were measured on the Seahorse XF24 analyzer for each cell line and changes versus untreated baseline are plotted. Error bars represent standard-deviation of means between experiments (N = 2–4). Each experiment consisted of 3–4 replicate wells per cell line with four serial measures performed on each well. p-values (Tukey-Kramer test) of statistically significant differences from CP-A are shown.

In response to 2,4-DNP, CP-C and CP-D had a lower increase in ECAR than CP-A (p<0.001 and p<10^−7^ respectively; Figure 3a and Table S4). In response to 2,4-DNP, CP-B and CP-D had a higher increase in OCR than CP-A (p<10^−7^ and p<0.05 respectively; Figure 3b and Table S4).

### CP-C and CP-D cell lines have the greatest modulation of OCR via the Crabtree effect

The Crabtree effect decreases oxidative phosphorylation in response to increasing glucose concentration and allows cancer and proliferating cells to adjust their energy metabolism depending on substrate availability. Since some BE cell lines displayed upregulated glycolysis while maintaining the ability to upregulate oxidative phosphorylation, we tested whether glycolytic cells displayed the Crabtree effect, another phenotype associated with progression to cancer.

**Figure 4:**
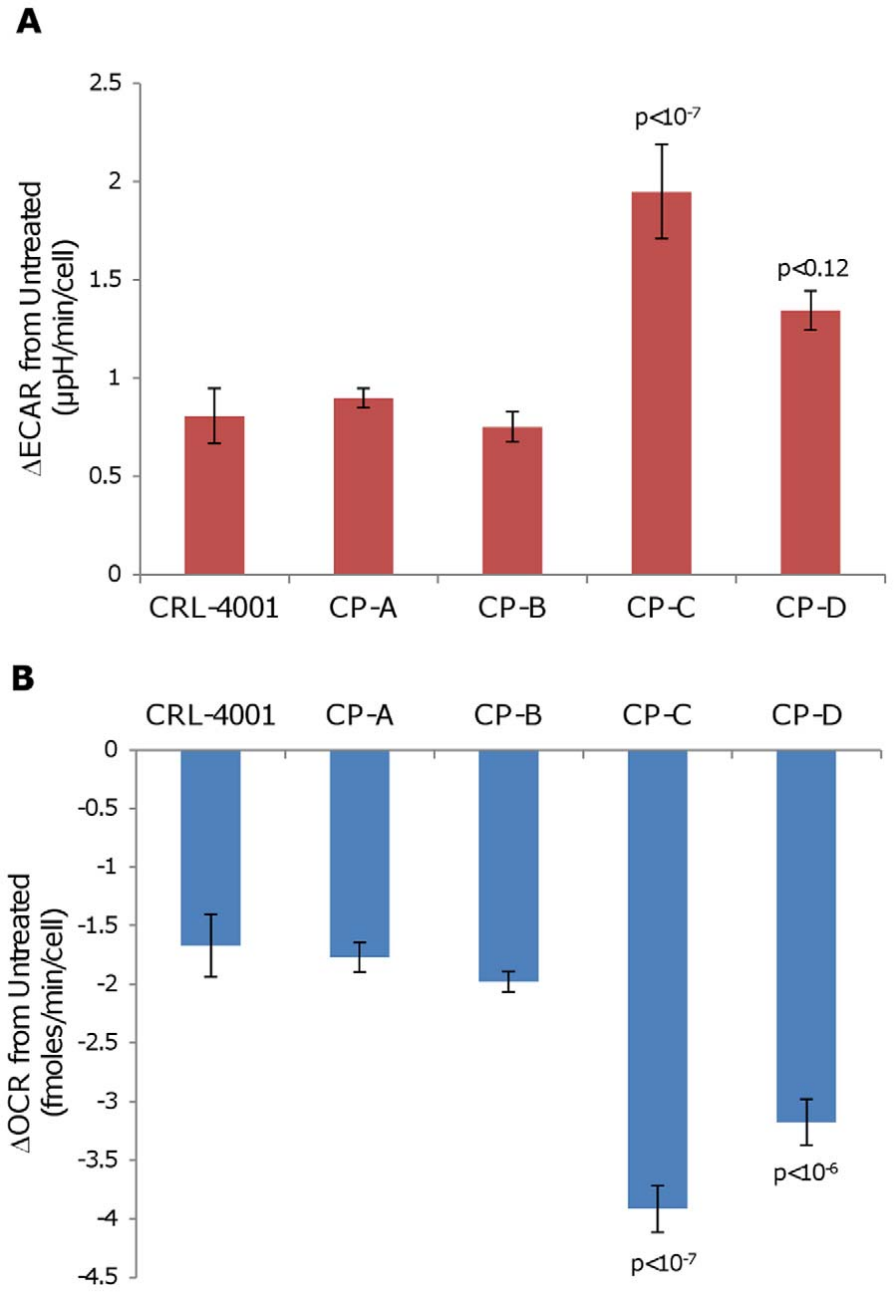
CP-C and CP-D demonstrate a stronger Crabtree effect response than other cell lines. Following the increase of glucose concentration in media from 0 mM to 5 mM, total (a) ECAR and (b) OCR were measured on the Seahorse XF24 analyzer for each cell line and changes versus untreated baseline are plotted. Error bars represent standard-deviation of means between experiments (N = 2–4). Each experiment consisted of 3–4 replicate wells per cell line with four serial measures performed on each well. p-values (Tukey-Kramer test) indicating significant differences from CP-A are shown.

In response to increased glucose concentrations from 0 mM to 5 mM, ECAR increased more in CP-C than in CP-A (p<10^−7^; Figure 4a and Table S5). ECAR in CP-D also increased more than in CP-A, but was not statistically significant (p<0.12). With the same treatment, OCR decreased more in CP-C and CP-D than in CP-A (p<10^−7^ and p<10^−6^, respectively; Figure 4b and Table S5). Subsequent addition of the mitochondrial uncoupler CCCP increased OCR in all cell lines, indicating that mitochondrial coupling was maintained.

### CP-B, CP-C and CP-D display high levels of genetic instability, but few alterations in energy metabolism genes

To determine if genetic changes exist in genes that may predict cell line differences in glycolysis and oxidative phosphorylation, the cell line nuclear genomes and mitochondrial genomes were analyzed using Illumina Omni-quad 1 M SNP arrays and Affymetrix MitoChip V.2 arrays, respectively (Text S2). Cell lines CP-B, CP-C and CP-D which were derived from patients with later stage BE had higher levels of chromosomal aberrations than CP-A (Figure S1 and Table S1). 30 nuclear-encoded genes that regulate glycolysis, oxidative phosphorylation, acid transport and hypoxia resistance were surveyed for somatic alterations; notably a single-copy of hypoxia-inducible factor 1α (HIF-1α) was deleted in both CP-C and CP-D (Table S6 and Text S2). CP-B and CP-C also had more single-copy deletions in nuclear-encoded mitochondrial genes than CP-A (Table S7).

We also examined if mitochondrial DNA mutations may predict cell line differences in glycolysis and oxidative phosphorylation. Multiple mtDNA mutations were detected in each of the cell lines, ranging from 2 in CP-D to 26 in CP-A (Table S8). None of these mutations were predictive of mitochondrial loss-of-function. Significant differences in mitochondrial mass were not observed between the cell lines (Figure S2).

## Discussion

This study provides the first evidence that cell lines derived from later-stages of the premalignant condition Barrett's esophagus have increased glycolysis compared to early-stage BE, while preserving mitochondrial function. Furthermore, we found that later-stage BE cell lines modulate their oxidative phosphorylation with an elevated Crabtree effect. Both of these phenotypic alterations are hallmarks of cancer metabolism and suggest that premalignant BE cell lines evolved their energy metabolism in response to variable oxygen and glucose levels.

We found that ECAR was highest in the three BE cell lines derived from patients with the highest genomic instability (CP-B, CP-C and CP-D) and lowest in the cell line derived from a patient with relatively more stable genome (CP-A), suggesting that a glycolytic phenotype was selected during the process of neoplastic progression. The BE cell line with the highest genomic instability, CP-D, was more glycolytic than the other BE cell lines, which was consistent with it having the greatest ECAR decrease following 2-DG addition and lowest ECAR increase following 2,4-DNP addition. However, mitochondrial function was conserved during CP-D's ‘glycolytic switch’ since it was able to compensate for glycolytic inhibition by upregulating oxidative phosphorylation. Although CP-C was more glycolytic than CP-A, it displayed a high level of OCR, which did not increase in response to glycolytic inhibition. This greater reliance on oxidative phosphorylation made CP-C more insensitive to glycolytic inhibition than CP-D. Similar to CP-D however, ECAR in CP-C was more insensitive to uncoupling by 2,4-DNP than CP-A, which is consistent with the higher baseline ECAR in CP-C. Insensitivity of glycolysis rates in CP-D to mitochondrial inhibition was also confirmed using the ATPase inhibitor oligomycin (Table S4). All cell lines maintained increased OCR levels in the absence of exogenous glutamine and glucose, indicating that mitochondrial upregulation is not specifically dependent on exogenous glutamine substrate (data not shown).

OCR in the BE cell lines did not correlate with ECAR, indicating that the changes in the two metabolic pathways evolved independently. The lack of OCR increase in CP-A, CP-B and CP-C cell lines in response to glycolytic inhibition suggests that ATP/ADP levels did not fall below a threshold that would stimulate mitochondrial activity, suggesting that oxidative phosphorylation supplied the majority of energy in these cell lines. Likewise, in response to 2,4-DNP-mediated uncoupling, OCR increases were not significantly different in CP-A and CP-C, and marginally higher in CP-D, suggesting that their mitochondria maintained a similar level of mitochondrial reserve capacity. This was also confirmed in an independent experiment in which oligomycin-treated BE cells had similar percentage OCR decreases, which corresponds to a similar contribution of ATP-generating coupled-respiration to total cellular OCR (Table S4). CP-B, the BE cell line which displayed the highest OCR, also displayed the highest OCR increase in response to 2,4-DNP, indicating that this cell line had the highest mitochondrial reserve capacity and/or utilized different available mitochondrial substrates. Increased mitochondrial reserve capacity has been shown to be dependent on substrate concentration in cancer cell lines but also differs based on mitochondrial enzyme activity in normal tissues [30], [31]. Despite evidence that would suggest mitochondrial differences between the BE cell lines, mitochondrial mass assessed by flow cytometric assays were not found to be different (Figures S2).

Finally, CP-C and CP-D cell lines demonstrated a greater change in OCR via Crabtree effect than the less glycolytic CP-A and CP-B cell lines, suggesting adaptation towards a more cancer-like phenotype. Increased Crabtree effect in BE cells predicts a greater survival advantage to adapt to conditions of glucose and oxygen fluctuation such as gastric reflux-induced ulceration or ischemia.

Difficulties associated with OE-33 cell adhesion prevented normalization of data collected for this EA cell line for direct comparison to the BE cell lines. However, based on normalization estimated from a limited data set, OE-33 had the highest levels of ECAR out of the cell lines tested and had percent ECAR that decreased as much as CP-D following 2-DG addition, indicating that it is a significantly glycolytic cell line (Text S1). OE-33 also had the highest levels of OCR and was sensitive to oligomycin, indicating a glycolytic cell line with functional mitochondria.

**Figure 5:**
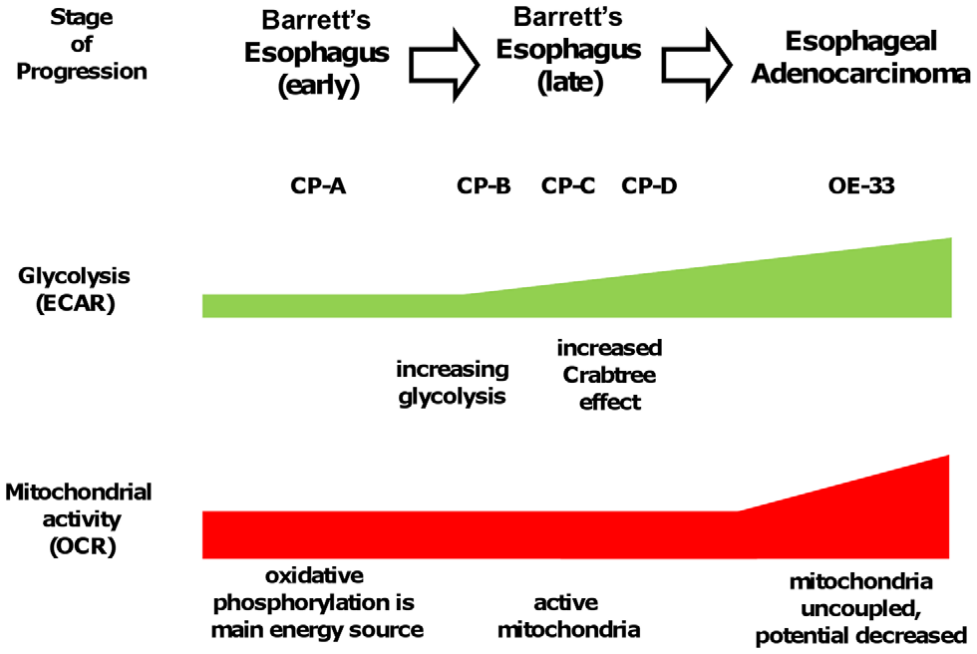
Barrett's esophagus progression to esophageal adenocarcinoma involves an intermediate metabolic stage with increased glycolysis and functional mitochondria. Early-stage BE cells (e.g. CP-A), rely mainly on mitochondrial oxidative phosphorylation for energy needs prior to the glycolytic increase which occurs in late-stage BE cells (CP-B, CP-C and CP-D), which demonstrate elevated ECAR (all) and the Crabtree effect (CP-C and CP-D). Finally in esophageal adenocarcinoma (OE-33) mitochondrial uncoupling occurs with increased OCR and glycolysis.

Given our findings, we propose a model in which BE begins with a metabolism which is largely dependent on oxidative phosphorylation, as seen in CP-A, but then progresses through a metabolic phenotype which is intermediate between normal and cancer (Figure 5). This intermediate stage displays increased glycolysis while mitochondria remain functional. As the cells progress, later stages of BE display a more pronounced Crabtree effect, which enables mitochondrial downregulation in response to substrate. Mitochondrial activity and uncoupling subsequently increase as the tissue progresses to EA.

Several mechanisms are likely to contribute to the metabolic changes in the BE cell lines. Both copies of TP53 are mutated and/or altered in CP-B, CP-C, and CP-D (Table S1), which may be of significance since loss of TP53 has been reported to upregulate glycolysis and downregulate mitochondrial activity to produce the Warburg effect in several cell lines [32], [33]. Notably the TP53-wild type BE cell line, CP-A, which is more representative of the genotype of BE patients that do not progress to cancer, demonstrated lower levels of glycolysis. A number of hypoxia-response genes have also been reported in BE tissue: glucose transporter Glut-1 [34] and pyruvate kinase isoform M2 [35], both known to be associated with elevated glycolysis, vascular endothelial growth factor (VEGF) and erythropoietin [20], which are upregulated by hypoxia-induced transcription factors HIF-1α and HIF-2α respectively. The genomic analysis performed in our study revealed that CP-C and CP-D have a single copy loss involving HIF-1α, however, the functional status of the remaining allele is unknown (Table S6). Overexpression of HIF-1α has been reported in other cancers [36], [37], [38] and may result from degradation-insensitive forms of this protein or increases in mTOR activity [39]. CP-D was also found to contain an amplified PHDGH gene (Human Cancer Genome Atlas), which is related to serine biogenesis and suggests an alternate use of the upregulated glycolytic pathway in this cell line [40]. The identification of a glycolytic phenotype in BE should bring to attention the increased importance of glycolytic and hypoxia-resistance pathways, which are routinely investigated in cancers but may also become altered earlier in progression.

Since endogenous reactive oxygen species (ROS) are produced by mitochondria, the mitochondrial genome is particularly susceptible to ROS-mediated damage. Although CP-A had the fewest nuclear genome alterations (Figure S1), it displayed the most mitochondrial genome mutations of all the cell lines (Table S7). Most of the CP-A mutations were heteroplasmic which indicates that multiple different clones were present. However, this diversity may also indicate that the predominantly oxidative phosphorylation metabolism in this cell line generates higher ROS levels, consistent with findings by Chen et al [41]. Remarkably, we found that the cell line displaying the most nuclear genome alterations, CP-D, had the fewest number of mitochondrial genome mutations (Table S7), consistent with CP-D maintaining active mitochondria and suggesting that CP-D was effective at suppressing ROS in the mitochondria and/or underwent a mitochondrial genetic sweep which shifted energy metabolism away from oxidative phosphorylation.

Although the Warburg and Crabtree effects have been commonly associated with cancer progression, higher glycolysis and Crabtree effect have also been observed in actively proliferating non-cancerous cells, such as stem cells and lymphocytes [8], [42]. Since BE undergoes frequent damage and repair, higher rates of growth in this tissue could be promoted by elevated glycolysis which, through the pentose-phosphate pathway, provides higher levels of anabolic substrates and NADPH, an important cofactor responsible for glutathione regeneration which suppresses ROS. Chen et al reported that CP-C had lower toxicity than CP-A to exogenous ROS species[41], although glutathione levels were not different and the differences in toxicity may be due to CP-A's more sensitive wild type p53, which has intact apoptotic signaling. Although we did not study ROS levels in the BE cell lines, altering metabolism may be a mechanism for preventing higher levels of mutation during BE progression. Interestingly, of the four patients from whom the cell lines were derived, the patient from whom the most mitochondrially-active BE cell line (CP-B) was developed was the only patient in whom EA was detected.

Given the preliminary nature of our findings, it is important to determine if the metabolic changes found in BE cell lines are also found in biopsies from patients. Although tissue culture is clearly a model system, all of the metabolic measurements of ECAR and OCR were done at confluency, a crude approximation of the structure of cells in tissue. Also, the BE cell lines in this study were immortalized using hTERT, avoiding potential metabolic effects of other immortalization methods that affect p53, Rb, and Myc activity such as SV40 large T antigen. However, since the BE and EA cell lines used in this study were derived from different patients, further analysis of longitudinally collected samples within the same patient would more directly address these questions. The most promising support for our findings *in vivo* are from BE tissue quantitative fluorescent imaging which displays elevated NAD(P)H levels representative of glycolytic tissue [19], suggesting that our observation in BE cell lines may translate to BE segments in patients.

## Supporting Information

Figure S1 **Barrett's esophagus cell lines CP-B, CP-C and CP-D display higher genome instability than CP-A.** Genome copy number alterations, relative to normal patient-derived diploid matched control (horizontal line), were plotted for each of the cell lines. CP-B, CP-C and CP-D display a large number of chromosomal alterations, compared to CP-A. Black dots represent moving averages of copy number; Gray dots represent individual locus copy number.
(TIF)

Figure S2 **Barrett's esophagus cell lines are not significantly different in mitochondrial mass.** Representative experiments are shown with (a) profiles of relative mitotracker green intensity (linear scale) for each of the cell lines, gated on live-fraction by Sytox Orange and G1-fraction by Hoechst 33342 staining; and (b) comparisons of mean mitotracker ratios from two repeat experiments. Error bars represent standard deviation between experiments (N = 2). Comparable results are obtained when non-G1 fractions are included in the analysis.
(TIF)

Table S1 **BE cell lines display genomic alterations that confer risk of progression (p16, p53) or are genetically unstable (FHIT, WWOX).** Deletions in genes that confer risk of progression to EA are reported. Mutations in p16 and TP53 were determined by Palanca-Wessels et al [15]. Number of somatic genome alterations (SGA) are shown categorized by type (CNLOH  =  copy number loss of heterozygosity, SCNA  =  somatic copy number alterations) and indicate the total amount of the genome affected by CNLOH or SCNA in each respective cell line. ^*^ The patient from whom CP-B was derived also had EA detected in the same endoscopy.
(DOCX)

Table S2 **Analysis of copy number alterations of genes involved glycolysis, oxidative phosphorylation and hypoxia regulation in BE cell lines.** Gene symbol is the human genome Gene Symbol for the gene. Copy number gains are marked as ‘+’ and single copy losses as ‘−’. No double copy losses were detected in the genes investigated. ^*^Note that prolyl 4-hydroxylases are inhibitors of HIF-1 mediated hypoxic resistance.
(DOCX)

Table S3 **Effects of 2-DG treatment on ECAR and OCR in cell lines.** The mean changes in ECAR and OCR after addition of 50 mM 2-DG compared to untreated baseline measured by Seahorse XF24 (N = 2–4). Abbreviations: SD = standard−deviation of means; p-value (Tukey-Kramer test) of statistically significant differences from CP-A are shown.
(DOCX)

Table S4 **Effects of 2,4-DNP and Oligomycin on ECAR and OCR in cell lines.** The mean changes in ECAR and OCR after addition of 50 µM 2,4-DNP compared to untreated baseline measured by Seahorse XF24 (N = 2–4). Abbreviations: SD = standard−deviation of means; p-value (Tukey-Kramer test) of statistically significant differences from CP-A are shown.
(DOCX)

Table S5 **Effects of glucose on changes in ECAR and OCR in cell lines via the Crabtree effect.** The mean changes in ECAR and OCR after addition of 5 mM glucose compared to glucose-free baseline measured by Seahorse XF24 (N = 2–4). Abbreviations: SD = standard−deviation of means; p-value (Tukey-Kramer test) of statistically significant differences from CP-A are shown.
(DOCX)

Table S6 **Analysis of copy number alterations of genes involved glycolysis, oxidative phosphorylation and hypoxia regulation in BE cell lines.** Gene symbol is the human genome Gene Symbol for the gene. Copy number gains are marked as ‘+’ and single copy losses as ‘−’. No double copy losses were detected in the genes investigated. ^*^Note that prolyl 4-hydroxylases are inhibitors of HIF-1 mediated hypoxic resistance.
(DOCX)

Table S7 **Summary of copy number alterations in nuclear-encoded mitochondrial genes.** 1023 mitochondrial genes were characterized for deletions, gains of copy number, and copy neutral loss of heterozygosity (LOH).
(DOCX)

Table S8 **Summary of mitochondrial mutations in BE derived cell lines.** Nucleotides for mutation locations and haplogroup designation based upon data from Mitomaster mitochondrial mutation database and analysis tool [26]. ^*^ indicates mutations that share their location with a known human mitochondrial polymorphism.
(DOCX)

Text S1 **BE and control cell line background and characterization.** This file contains the background and characterization of four BE cell lines derived from BE segments, three genetically-stable control cell lines, and cell line derived from an adenocarcinoma.
(DOCX)

Text S2 **Nuclear and mitochondrial genome characterization.** This file contains additional details from the nuclear and mitochondrial genome analyses performed.
(DOCX)
