# Supplementary figures and images for: Warburg and Crabtree Effects in Premalignant Barrett's Esophagus Cell Lines with Active Mitochondria
Source: PLoS One. 2013 Feb 27;8(2):e56884. doi: 10.1371/journal.pone.0056884 (PMC3584058; doi:10.1371/journal.pone.0056884)

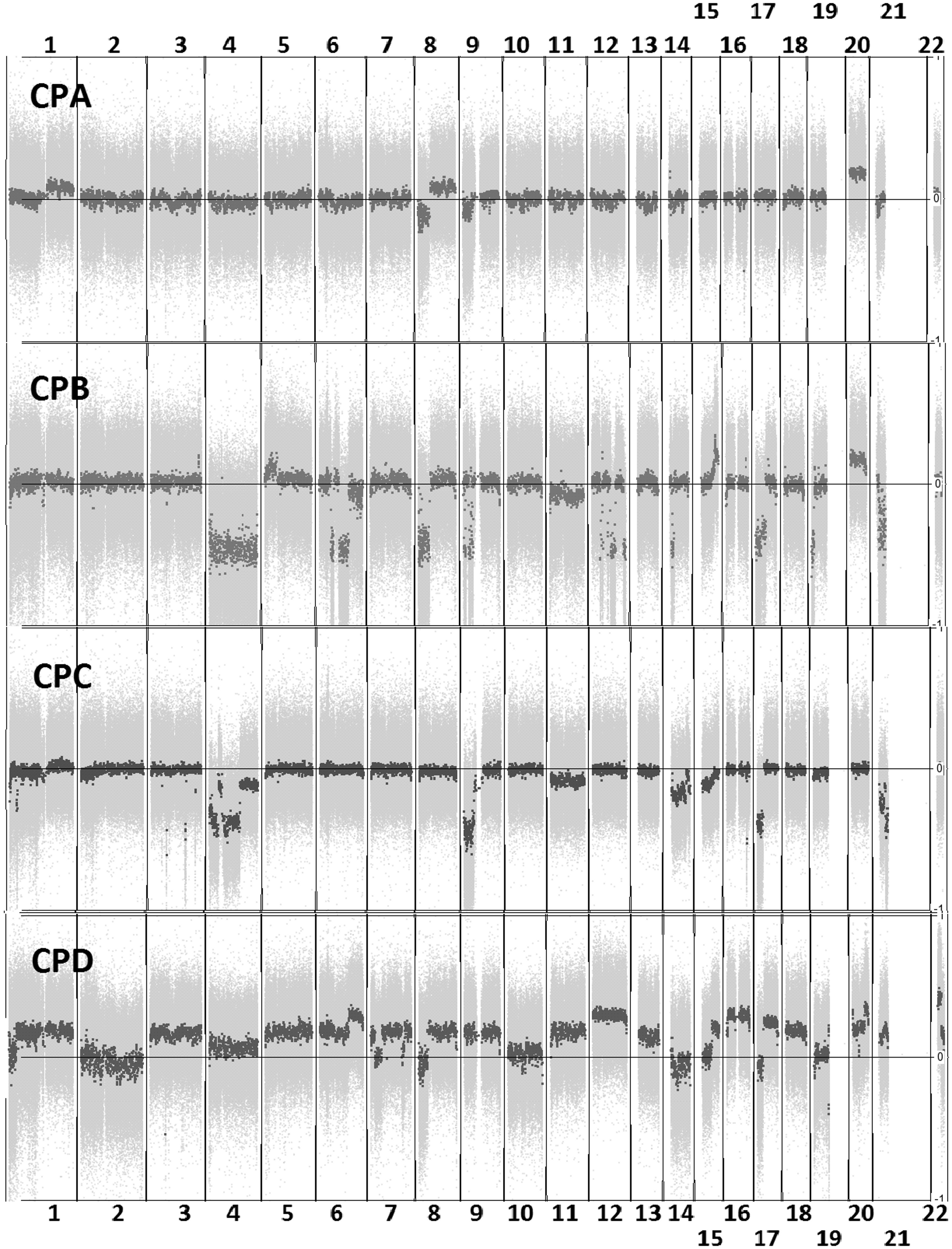

Supplement: Figure S1 — Barrett's esophagus cell lines CP-B, CP-C and CP-D display higher genome instability than CP-A. Genome copy number alterations, relative to normal patient-derived diploid matched control (horizontal line), were plotted for each of the cell lines. CP-B, CP-C and CP-D display a large number of chromosomal alterations, compared to CP-A. Black dots represent moving averages of copy number; Gray dots represent individual locus copy number. (TIF) [file pone.0056884.s001.tif]

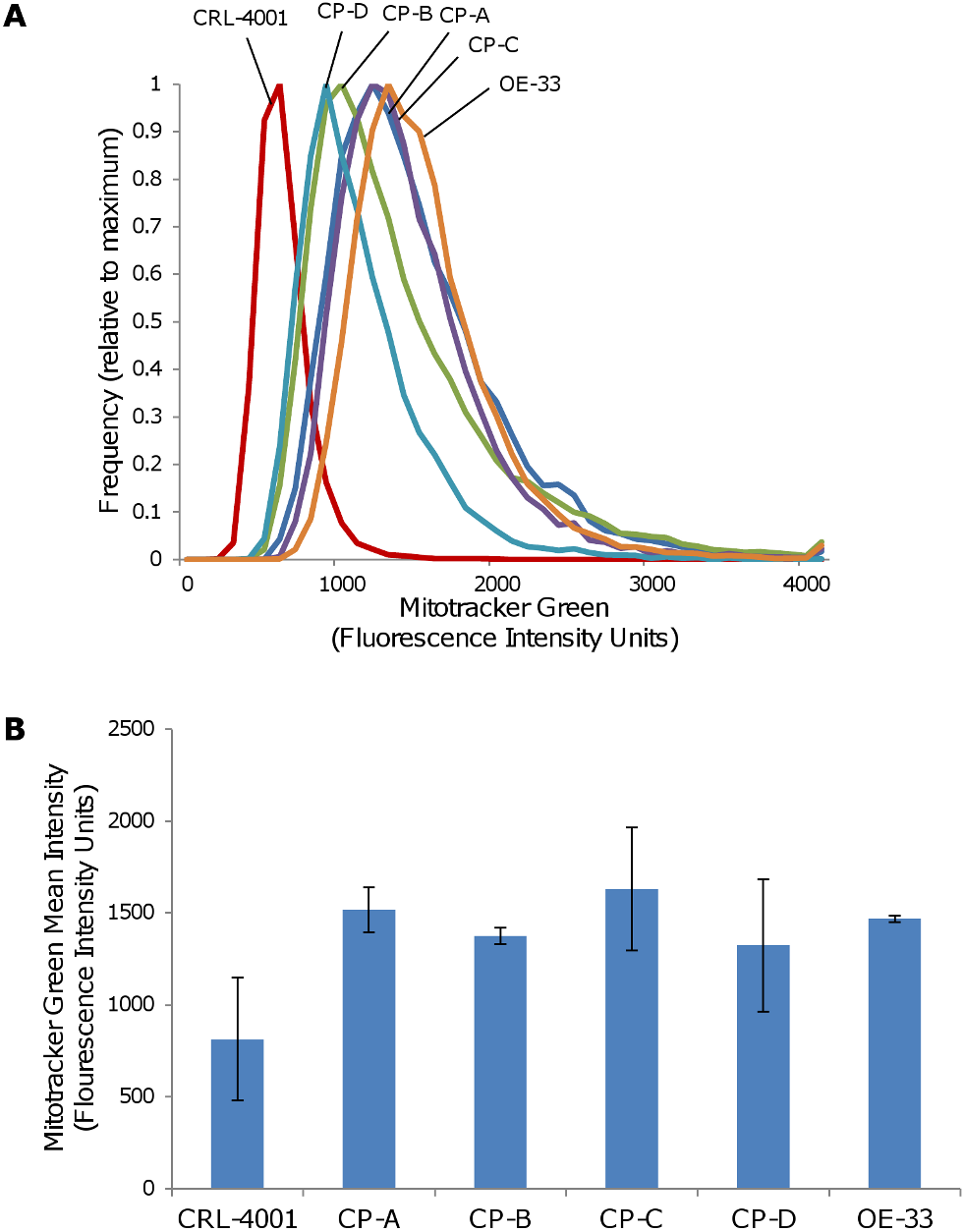

Supplement: Figure S2 — Barrett's esophagus cell lines are not significantly different in mitochondrial mass. Representative experiments are shown with (a) profiles of relative mitotracker green intensity (linear scale) for each of the cell lines, gated on live-fraction by Sytox Orange and G1-fraction by Hoechst 33342 staining; and (b) comparisons of mean mitotracker ratios from two repeat experiments. Error bars represent standard deviation between experiments (N = 2). Comparable results are obtained when non-G1 fractions are included in the analysis. (TIF) [file pone.0056884.s002.tif]
