# Supplementary material for: Warburg and Crabtree Effects in Premalignant Barrett's Esophagus Cell Lines with Active Mitochondria
Source: PLoS One. 2013 Feb 27;8(2):e56884. doi: 10.1371/journal.pone.0056884 (PMC3584058; doi:10.1371/journal.pone.0056884)
Supplement: Table S1 — BE cell lines display genomic alterations that confer risk of progression (p16, p53) or are genetically unstable (FHIT, WWOX). Deletions in genes that confer risk of progression to EA are reported. Mutations in p16 and TP53 were determined by Palanca-Wessels et al [15]. Number of somatic genome alterations (SGA) are shown categorized by type (CNLOH = copy number loss of heterozygosity, SCNA = somatic copy number alterations) and indicate the total amount of the genome affected by CNLOH or SCNA in each respective cell line. * The patient from whom CP-B was derived also had EA detected in the same endoscopy. (DOCX) [file pone.0056884.s003.docx]

**Table S1: Status of BE cell line genes that indicate risk of progression and somatic genome alterations (SGA).**

| \| Cell Line \| Patient Diagnosis \| Gene deletions \| \| \| \| Somatic Genomic Alterations \| \| \| \| --- \| --- \| --- \| --- \| --- \| --- \| --- \| --- \| --- \| \|  \|  \| p16 \| p53 \| FHIT \| WWOX \| CNLOH SGA (Mb) \| SCNA SGA (Mb) \| Combined SGA (Mb) \| \| CP-A \| Negative for dysplasia \| double deletion \| wild type \| double deletion \| double deletion \| 126 \| 229 \| 355 \| \| CP-B \| High-grade dysplasia* \| single deletion, mutated \| single deletion, mutated \| double deletion \| amplification \| 66 \| 527 \| 593 \| \| CP-C \| High-grade dysplasia \| single deletion \| single deletion, mutated \| double deletion \| double deletion \| 15 \| 552 \| 567 \| \| CP-D \| High-grade dysplasia \| no deletion \| no deletion, mutated \| single deletion \| single deletion \| 373 \| 2067 \| 2440 \| |  |  |  |  |  |  |  |
| --- | --- | --- | --- | --- | --- | --- | --- | --- | --- | --- | --- | --- | --- | --- | --- | --- | --- | --- | --- | --- | --- | --- | --- | --- | --- | --- | --- | --- | --- | --- | --- | --- | --- | --- | --- | --- | --- | --- | --- | --- | --- | --- | --- | --- | --- | --- | --- | --- | --- | --- | --- | --- | --- | --- | --- | --- | --- | --- | --- | --- | --- |
