# Supplementary material for: Warburg and Crabtree Effects in Premalignant Barrett's Esophagus Cell Lines with Active Mitochondria
Source: PLoS One. 2013 Feb 27;8(2):e56884. doi: 10.1371/journal.pone.0056884 (PMC3584058; doi:10.1371/journal.pone.0056884)
Supplement: Table S2 — Analysis of copy number alterations of genes involved glycolysis, oxidative phosphorylation and hypoxia regulation in BE cell lines. Gene symbol is the human genome Gene Symbol for the gene. Copy number gains are marked as ‘+’ and single copy losses as ‘−’. No double copy losses were detected in the genes investigated. *Note that prolyl 4-hydroxylases are inhibitors of HIF-1 mediated hypoxic resistance. (DOCX) [file pone.0056884.s004.docx]

**Table S2: BE cell lines have significant differences in ECAR and OCR.**

|  | ECAR  (µpH/min/cell) | | | OCR_Total_  (fmoles/min/cell) | | | OCR_OxPhos_  (fmoles/min/cell) | |
| --- | --- | --- | --- | --- | --- | --- | --- | --- |
| Cell line | mean | SD | p-value | mean | SD | p-value | mean | SD |
| CRL-4001 | 1.4 | 0.2 | n.s. | 4.9 | 0.2 | n.s. | 3.6 | 0.2 |
| CP-A | 1.1 | 0.1 | - | 4.1 | 0.3 | - | 2.7 | 0.3 |
| CP-B | 1.6 | 0.1 | n.s. | 6.7 | 0.9 | <10^-7^ | 4.2 | 0.9 |
| CP-C | 2.1 | 0.1 | <0.001 | 5.7 | 0.3 | <0.001 | 3.4 | 0.3 |
| CP-D | 2.2 | 0.2 | <10^-7^ | 3.7 | 0.8 | n.s. | 2.1 | 0.8 |
