# Supplementary material for: Warburg and Crabtree Effects in Premalignant Barrett's Esophagus Cell Lines with Active Mitochondria
Source: PLoS One. 2013 Feb 27;8(2):e56884. doi: 10.1371/journal.pone.0056884 (PMC3584058; doi:10.1371/journal.pone.0056884)
Supplement: Table S3 — Effects of 2-DG treatment on ECAR and OCR in cell lines. The mean changes in ECAR and OCR after addition of 50 mM 2-DG compared to untreated baseline measured by Seahorse XF24 (N = 2–4). Abbreviations: SD = standard−deviation of means; p-value (Tukey-Kramer test) of statistically significant differences from CP-A are shown. (DOCX) [file pone.0056884.s005.docx]

**Table S3: Effects of 2-DG treatment on ECAR and OCR in cell lines.**

|  | ΔECAR from untreated baseline (µpH/min/cell) | | | ΔECAR from untreated baseline (% change) | ΔOCR from untreated baseline (fMoles/min/cell) | | | ΔOCR from untreated baseline (% change) |
| --- | --- | --- | --- | --- | --- | --- | --- | --- |
| Cell line | mean | SD | p-value |  | mean | SD | p-value |  |
| CRL-4001 | -1.19 | 0.07 | <10^-4^ | -66 | -0.37 | 0.22 | n.s. | -7 |
| CP-A | -0.81 | 0.05 | - | -67 | -0.26 | 0.07 | - | -6 |
| CP-B | -0.91 | 0.04 | n.s. | -60 | -0.32 | 0.10 | n.s. | -6 |
| CP-C | -1.18 | 0.05 | <10^-4^ | -54 | -0.21 | 0.15 | n.s. | -4 |
| CP-D | -1.84 | 0.05 | <10^-7^ | -70 | 0.58 | 0.15 | <0.01 | +21 |
