# Supplementary material for: Warburg and Crabtree Effects in Premalignant Barrett's Esophagus Cell Lines with Active Mitochondria
Source: PLoS One. 2013 Feb 27;8(2):e56884. doi: 10.1371/journal.pone.0056884 (PMC3584058; doi:10.1371/journal.pone.0056884)
Supplement: Table S4 — Effects of 2,4-DNP and Oligomycin on ECAR and OCR in cell lines. The mean changes in ECAR and OCR after addition of 50 µM 2,4-DNP compared to untreated baseline measured by Seahorse XF24 (N = 2–4). Abbreviations: SD = standard−deviation of means; p-value (Tukey-Kramer test) of statistically significant differences from CP-A are shown. (DOCX) [file pone.0056884.s006.docx]

**Table S4: Effects of 2,4-DNP and Oligomycin on ECAR and OCR in cell lines.**

|  | ΔECAR from untreated baseline following 2,4-DNP  (µpH/min/cell) | | | ΔECAR from untreated baseline following 2,4-DNP (% change) | ΔOCR from untreated baseline following 2,4-DNP (fMoles/min/cell) | | | ΔOCR from untreated baseline following 2,4-DNP (% change) | ΔECAR from untreated baseline following oligomycin  (% change) | ΔOCR from untreated baseline following oligomycin  (% change) |
| --- | --- | --- | --- | --- | --- | --- | --- | --- | --- | --- |
| Cell line | mean | SD | p-value |  | mean | SD | p-value |  |  |  |
| CRL-4001 | 0.76 | 0.05 | n.s. | +73 | 3.0 | 0.2 | n.s. | +69 | +42 | -73 |
| CP-A | 0.80 | 0.03 | - | +79 | 6.6 | 0.3 | - | +156 | +59 | -66 |
| CP-B | 0.93 | 0.07 | n.s. | +53 | 21.3 | 2.0 | <10^-7^ | +271 | +86 | -63 |
| CP-C | 0.49 | 0.05 | <0.001 | +26 | 6.8 | 0.3 | n.s. | +122 | +81 | -60 |
| CP-D | 0.23 | 0.04 | <10^-7^ | +8 | 11.0 | 1.4 | <0.05 | +157 | +30 | -57 |
