# Supplementary material for: Warburg and Crabtree Effects in Premalignant Barrett's Esophagus Cell Lines with Active Mitochondria
Source: PLoS One. 2013 Feb 27;8(2):e56884. doi: 10.1371/journal.pone.0056884 (PMC3584058; doi:10.1371/journal.pone.0056884)
Supplement: Table S5 — Effects of glucose on changes in ECAR and OCR in cell lines via the Crabtree effect. The mean changes in ECAR and OCR after addition of 5 mM glucose compared to glucose-free baseline measured by Seahorse XF24 (N = 2–4). Abbreviations: SD = standard−deviation of means; p-value (Tukey-Kramer test) of statistically significant differences from CP-A are shown. (DOCX) [file pone.0056884.s007.docx]

**Table S5: Effects of glucose on changes in ECAR and OCR in cell lines via the Crabtree effect**.

|  | ΔECAR from baseline  (µpH/min/cell) | | | ΔECAR from baseline  (% change) | ΔOCR from baseline (fMoles/min/cell) | | | ΔOCR from baseline  (% change) |
| --- | --- | --- | --- | --- | --- | --- | --- | --- |
| Cell line | mean | SD | p-value |  | mean | SD | p-value |  |
| CRL-4001 | 0.81 | 0.14 | n.s. | +104 | -1.7 | 0.3 | n.s. | -45 |
| CP-A | 0.90 | 0.05 | - | +130 | -1.8 | 0.1 | - | -34 |
| CP-B | 0.75 | 0.08 | n.s. | +67 | -2.0 | 0.1 | n.s. | -25 |
| CP-C | 1.95 | 0.24 | <10^-7^ | +196 | -3.9 | 0.2 | <10^-7^ | -60 |
| CP-D | 1.34 | 0.10 | <0.12 | +132 | -3.2 | 0.2 | <10^-6^ | -53 |
