# Supplementary material for: Warburg and Crabtree Effects in Premalignant Barrett's Esophagus Cell Lines with Active Mitochondria
Source: PLoS One. 2013 Feb 27;8(2):e56884. doi: 10.1371/journal.pone.0056884 (PMC3584058; doi:10.1371/journal.pone.0056884)
Supplement: Table S6 — Analysis of copy number alterations of genes involved glycolysis, oxidative phosphorylation and hypoxia regulation in BE cell lines. Gene symbol is the human genome Gene Symbol for the gene. Copy number gains are marked as ‘+’ and single copy losses as ‘−’. No double copy losses were detected in the genes investigated. *Note that prolyl 4-hydroxylases are inhibitors of HIF-1 mediated hypoxic resistance. (DOCX) [file pone.0056884.s008.docx]

**Table S6: Analysis of copy number alterations of genes involved glycolysis, oxidative phosphorylation and hypoxia regulation in BE cell lines.**

| Gene Investigated | | | Cell line | | | |
| --- | --- | --- | --- | --- | --- | --- |
| Gene Symbol | Gene name | Gene function | CP-A | CP-B | CP-C | CP-D |
| HK1 | hexokinase 1 | Glycolysis |  |  |  |  |
| HK2 | hexokinase 2 | Glycolysis |  |  |  |  |
| LDHA | lactate dehydrogenase A | Glycolysis |  |  | - | + |
| LDHB | lactate dehydrogenase B | Glycolysis | + |  |  | + |
| LDHC | lactate dehydrogenase C | Glycolysis |  |  | - | + |
| LDHD | lactate dehydrogenase D | Glycolysis |  |  |  | + |
| PFKL | phosphofructokinase, liver | Glycolysis |  |  | - | + |
| PFKM | phosphofructokinase, muscle | Glycolysis |  |  |  | + |
| PFKP | phosphofructokinase, platelet | Glycolysis |  |  |  |  |
| PGK1 | phosphoglycerate kinase 1 | Glycolysis |  |  |  | + |
| PGK2 | phosphoglycerate kinase 2 | Glycolysis | + | - |  | + |
| PKM2 | pyruvate kinase, muscle | Glycolysis |  |  |  | + |
| SLC2A1 | solute carrier family 2 (facilitated glucose transporter), member 1 | Glycolysis |  |  |  | + |
| PDHA2 | pyruvate dehydrogenase (lipoamide) alpha 2 | Regulation of Oxidative Phosphorylation |  | - | - |  |
| PDHB | pyruvate dehydrogenase (lipoamide) beta | Regulation of Oxidative Phosphorylation |  |  |  |  |
| PDHX | pyruvate dehydrogenase complex, component X | Regulation of Oxidative Phosphorylation |  |  | - | + |
| SCO2 | SCO cytochrome oxidase deficient homolog 2 (yeast) | Regulation of Oxidative Phosphorylation |  |  |  | + |
| ATPIF1 | ATPase inhibitory factor 1 | Regulation of ATPase |  |  |  | - |
| CA9 | carbonic anhydrase IX | Acid transporter |  |  | - | + |
| CA12 | carbonic anhydrase XII | Acid transporter |  |  |  |  |
| SLC9A1 | solute carrier family 9 (sodium/hydrogen exchanger), member 1 | Acid transporter |  |  | - |  |
| EPO | erythropoietin | Hypoxia resistance |  |  |  | + |
| HIF1A | hypoxia inducible factor 1, alpha subunit | Hypoxia resistance |  |  | - | - |
| HIF2A/EPAS1 | endothelial PAS domain protein 1 | Hypoxia resistance |  |  |  |  |
| HIF3A | hypoxia inducible factor 3, alpha subunit | Hypoxia resistance |  |  |  |  |
| P4HA1* | prolyl 4-hydroxylase, alpha polypeptide I | Hypoxia resistance |  |  |  |  |
| P4HA2* | prolyl 4-hydroxylase, alpha polypeptide II | Hypoxia resistance | + |  |  | + |
| P4HA3* | prolyl 4-hydroxylase, alpha polypeptide III | Hypoxia resistance |  |  | - | + |
| VEGFA | vascular endothelial growth factor A | Hypoxia resistance |  |  |  | + |
| VEGFB | vascular endothelial growth factor B | Hypoxia resistance |  |  | - | + |
| VEGFC | vascular endothelial growth factor C | Hypoxia resistance |  | - | - |  |
| VHL | von Hippel-Lindau tumor suppressor | Hypoxia resistance |  |  |  |  |
