# Supplementary material for: Warburg and Crabtree Effects in Premalignant Barrett's Esophagus Cell Lines with Active Mitochondria
Source: PLoS One. 2013 Feb 27;8(2):e56884. doi: 10.1371/journal.pone.0056884 (PMC3584058; doi:10.1371/journal.pone.0056884)
Supplement: Table S7 — Summary of copy number alterations in nuclear-encoded mitochondrial genes. 1023 mitochondrial genes were characterized for deletions, gains of copy number, and copy neutral loss of heterozygosity (LOH). (DOCX) [file pone.0056884.s009.docx]

**Table S7: Summary of copy number alterations in nuclear-encoded mitochondrial genes.**

| Cell line | Single-copy deletions | Double deletions | Gains | Copy-neutral loss of heterozygosity |
| --- | --- | --- | --- | --- |
| CP-A | 10 | 0 | 211 | 61 |
| CP-B | 174 | 1 | 33 | 47 |
| CP-C | 156 | 0 | 6 | 1 |
| CP-D | 11 | 0 | 667 | 113 |
