# Supplementary material for: Warburg and Crabtree Effects in Premalignant Barrett's Esophagus Cell Lines with Active Mitochondria
Source: PLoS One. 2013 Feb 27;8(2):e56884. doi: 10.1371/journal.pone.0056884 (PMC3584058; doi:10.1371/journal.pone.0056884)
Supplement: Table S8 — Summary of mitochondrial mutations in BE derived cell lines. Nucleotides for mutation locations and haplogroup designation based upon data from Mitomaster mitochondrial mutation database and analysis tool [26]. * indicates mutations that share their location with a known human mitochondrial polymorphism. (DOCX) [file pone.0056884.s010.docx]

**Table S8. Summary of mitochondrial mutations in BE derived cell lines.**

| Cell line | Haplogroup | Mitochondrial mutation (basepair) | Mitochondrial mutation | Gene affected |
| --- | --- | --- | --- | --- |
| CP-A | H2 | 72^*^ | a>g | D-Loop |
| CP-A | H2 | 194 | t>c | D-Loop |
| CP-A | H2 | 1437^*^ | a>g | 12S ribosomal RNA |
| CP-A | H2 | 2580 | a>g | 16S ribosomal RNA |
| CP-A | H2 | 2705^*^ | a>g | 16S ribosomal RNA |
| CP-A | H2 | 4644 | t>c | NADH dehydrogenase subunit 0 |
| CP-A | H2 | 4767^*^ | a>g | NADH dehydrogenase subunit 1 |
| CP-A | H2 | 4809 | a>g | NADH dehydrogenase subunit 2 |
| CP-A | H2 | 5997 | t>c | Cytochrome c oxidase subunit I |
| CP-A | H2 | 6045 | a>g | Cytochrome c oxidase subunit I |
| CP-A | H2 | 6144 | a>g | Cytochrome c oxidase subunit I |
| CP-A | H2 | 6258 | g>a | Cytochrome c oxidase subunit I |
| CP-A | H2 | 7026 | c>t | Cytochrome c oxidase subunit I |
| CP-A | H2 | 9068 | t>g | ATP synthase F0 subunit 6 |
| CP-A | H2 | 10596 | a>g | NADH dehydrogenase subunit 4L |
| CP-A | H2 | 10905 | t>c | NADH dehydrogenase subunit 4 |
| CP-A | H2 | 11007 | t>c | NADH dehydrogenase subunit 5 |
| CP-A | H2 | 11330 | c>t | NADH dehydrogenase subunit 6 |
| CP-A | H2 | 11465 | a>g | NADH dehydrogenase subunit 7 |
| CP-A | H2 | 11717^*^ | g>a | NADH dehydrogenase subunit 8 |
| CP-A | H2 | 12306 | a>g | tRNA leucine2 |
| CP-A | H2 | 12370 | g>a | NADH dehydrogenase subunit 5 |
| CP-A | H2 | 14618 | c>t | NADH dehydrogenase subunit 6 |
| CP-A | H2 | 14764^*^ | c>t | Cytochrome b |
| CP-A | H2 | 14864 | c>t | Cytochrome b |
| CP-A | H2 | 15691 | t>c | Cytochrome b |
| CB-B | H1 | 151 | t>c | D-Loop |
| CB-B | H1 | 6717 | t>c | Cytochrome c oxidase subunit I |
| CB-B | H1 | 8243 | a>g | Cytochrome c oxidase subunit II |
| CB-B | H1 | 12616 | g>a | NADH dehydrogenase subunit 6 |
| CP-C | U | 145 | t>c | D-Loop |
| CP-C | U | 152 | a>g | D-Loop |
| CP-C | U | 194 | t>c | D-Loop |
| CP-C | U | 1718 | g>a | 16S ribosomal RNA |
| CP-C | U | 2180 | a>g | 16S ribosomal RNA |
| CP-C | U | 3916 | g>a | NADH Dehydrogenase subunit 1 |
| CP-C | U | 6219 | t>c | Cytochrome c oxidase subunit I |
| CP-C | U | 6369 | c>t | Cytochrome c oxidase subunit I |
| CP-C | U | 12649 | g>a | NADH dehydrogenase subunit 7 |
| CP-C | U | 12703 | c>t | NADH dehydrogenase subunit 8 |
| CP-C | U | 13964 | a>g | NADH dehydrogenase subunit 9 |
| CP-C | U | 14468 | t>c | NADH dehydrogenase subunit 5 |
| CP-C | U | 14770 | c>t | Cytochrome b |
| CP-C | U | 15312 | g>a | Cytochrome b |
| CP-C | U | 16221 | c>t | D-Loop |
| CP-C | U | 16253 | g>a | D-Loop |
| CP-C | U | 16276 | c>t | D-Loop |
| CP-C | U | 16292 | c>t | D-Loop |
| CP-D | H1 | 256 | a>g | D-Loop |
| CP-D | H1 | 476 | t>c | D-Loop |
