# Supplementary material for: Warburg and Crabtree Effects in Premalignant Barrett's Esophagus Cell Lines with Active Mitochondria
Source: PLoS One. 2013 Feb 27;8(2):e56884. doi: 10.1371/journal.pone.0056884 (PMC3584058; doi:10.1371/journal.pone.0056884)
Supplement: Text S1 — BE and control cell line background and characterization. This file contains the background and characterization of four BE cell lines derived from BE segments, three genetically-stable control cell lines, and cell line derived from an adenocarcinoma. (DOCX) [file pone.0056884.s011.docx]

**Text S1: BE and control cell line background and characterization.**

**BE cell lines.** The BE cell lines were previously shown to contain the same genetic lesions as BE segments of patients from whom the lines were derived [[1](file:///C:\Users\martin\Desktop\Work\Thesis\Chapter1\for%20DHockenbery\Text%20S1.docx#_ENREF_1)]. CP-A is the only cell line derived from an individual without dysplasia and contains a wild-type p53 (Table S1). CP-B, CP-C and CP-D cell lines were derived from patients with high grade dysplasia; all contain bi-allelic p16 or p53 alterations and have greater genomic alterations, with CP-D displaying the most genetic instability and multiple aneuploid sub-populations. Cell lines were started at low passage numbers to avoid development of extra aneuploid sub-populations, and were refreshed from stock after 20 passages, or upon detection of changes in DNA content.

The research participant from whom the CP-A cell line was developed never developed high-grade dysplasia or esophageal adenocarcinoma and has been active in the Seattle Barrett’s Esophagus study for 21 years. The research participant from whom the CP-B cell line was developed had an 11cm BE segment with a visible nodule. A total of 57 biopsies were evaluated histologically in the BE segment and EA was detected in 4 of 13 biopsies taken at the level with the visible nodule.  The biopsies taken for tissue culture were collected from the BE mucosa at a level different from the one at which cancer was diagnosed. The research participant from whom the CP-C cell line was developed was followed with high-grade dysplasia for 6.3 years and opted for an esophagectomy for high-grade dysplasia and died of mesothelioma 11.9 years later. The research participant from whom the CP-D cell line was developed was followed with high-grade dysplasia for 8.3 years and died of natural causes (pneumonia) 9.6 years later.

**Genetically stable control cell line.** As potential esophageal squamous control cell lines, we considered cell lines EPC-2 and HET-1A. EPC-2, an esophageal squamous cell line was initially proposed as a normal cell line control since it was reported to have wild-type p16 and p53 loci [[2](file:///C:\Users\martin\Desktop\Work\Thesis\Chapter1\for%20DHockenbery\Text%20S1.docx#_ENREF_2)]. However, the cell line that was provided from the Carlo Maley lab (UCSF) was abandoned after it was found to be aneuploid by flow cytometric DNA content quantitation (data not shown). HET-1A was not used since it was transformed with simian virus 40 large T-antigen, which inhibits Rb and p53 activity, a modulator of glycolytic and oxidative phosphorylation in cells [[3](file:///C:\Users\martin\Desktop\Work\Thesis\Chapter1\for%20DHockenbery\Text%20S1.docx#_ENREF_3)]. Recently, HET-1A has also been reported to form irregular dysplastic epithelial organotypic culture by Underwood et al (Underwood et al, 2010), further indicating that this cell line is not suitable as a normal esophageal control.

Since an esophageal squamous control cell line was not available, in this study we decided to use CRL-4001/BJ-5ta, a genetically stable hTERT-immortalized foreskin fibroblast cell line, which is guaranteed by ATCC to display genetic stability for up to ten passages (Geron Corporation, ATCC). Although it has been shown that fibroblasts and keratinocytes typically display different energy metabolism characteristics [[4](file:///C:\Users\martin\Desktop\Work\Thesis\Chapter1\for%20DHockenbery\Text%20S1.docx#_ENREF_4)], this cell line was used as an inter-experimental control due to its genetic stability, and it remained phenotypically consistent across our experiments.

**Adenocarcinoma cell line.** OE-33 was established from a Barrett’s associated adenocarcinoma of the distal esophagus and was chosen as an adenocarcinoma cell line control in this study [[5](file:///C:\Users\martin\Desktop\Work\Thesis\Chapter1\for%20DHockenbery\Text%20S1.docx#_ENREF_5)]. OE-33 is a hyperploid p53-inactive cell line with high mitotic index and was recently authenticated by Boonstra et al [[6](file:///C:\Users\martin\Desktop\Work\Thesis\Chapter1\for%20DHockenbery\Text%20S1.docx#_ENREF_6),[7](file:///C:\Users\martin\Desktop\Work\Thesis\Chapter1\for%20DHockenbery\Text%20S1.docx#_ENREF_7)].

Difficulties associated with cell adhesion prevented normalization of the EA cell line OE-33 and direct comparison to the BE cell lines. However based on cell counts obtained from a limited set of wells, we estimate that OE-33 was the most glycolytic out of the cell lines tested with ECAR up to four-fold higher compared to the other cell lines (4.1 µpH/min/cell). It also had the highest levels of oxidative phosphorylation with OCR five-fold higher compared to the other cell lines (27.6 fmol/min/cell). This higher OCR suggests a glycolytic cell line with 1) increased oxidative phosphorylation and/or 2) mitochondrial uncoupling. OCR in OE-33 was also highly decreased following treatment with ATPase inhibitor oligomycin, suggesting that mitochondria are involved in ATP synthesis in this cell line.

In response to 2-DG, percent ECAR decreased in OE-33 to levels similar to CP-D; however contrary to other cell lines, OCR in OE-33 decreased suggesting that the rate of pyruvate production from glycolysis is the rate limiting step for oxidative phosphorylation in this cell line (data not shown). In response to 2,4-DNP uncoupling, OE-33’s smaller increase in percent OCR than in the other BE cell lines suggests that mitochondria are already partially uncoupled (data not shown). Altogether, OE-33 displays characteristics of a glycolytic cancer cell line with active but partially uncoupled mitochondria. Thus, consistent with numerous other groups’ findings that mitochondria maintain their function in several cancers [[8](file:///C:\Users\martin\Desktop\Work\Thesis\Chapter1\for%20DHockenbery\Text%20S1.docx#_ENREF_8)],[[9](file:///C:\Users\martin\Desktop\Work\Thesis\Chapter1\for%20DHockenbery\Text%20S1.docx#_ENREF_9)],[[10](file:///C:\Users\martin\Desktop\Work\Thesis\Chapter1\for%20DHockenbery\Text%20S1.docx#_ENREF_10)], in the EA cell line that we examined, mitochondria remained functional while glycolysis was upregulated. In response to elevated glucose, OE-33 did not have percent ECAR and percent OCR increases that were significantly different compared to CRL-4001, CP-A, or CP-B; however absolute changes in OCR were estimated to be significantly higher than in other cell lines.

1. Palanca-Wessels MC, Barrett MT, Galipeau PC, Rohrer KL, Reid BJ, et al. (1998) Genetic analysis of long-term Barrett's esophagus epithelial cultures exhibiting cytogenetic and ploidy abnormalities. Gastroenterology 114: 295-304.

2. Harada H, Nakagawa H, Oyama K, Takaoka M, Andl CD, et al. (2003) Telomerase induces immortalization of human esophageal keratinocytes without p16INK4a inactivation. Molecular cancer research : MCR 1: 729-738.

3. Matoba S, Kang JG, Patino WD, Wragg A, Boehm M, et al. (2006) p53 regulates mitochondrial respiration. Science 312: 1650-1653.

4. Hornig-Do HT, von Kleist-Retzow JC, Lanz K, Wickenhauser C, Kudin AP, et al. (2007) Human epidermal keratinocytes accumulate superoxide due to low activity of Mn-SOD, leading to mitochondrial functional impairment. The Journal of investigative dermatology 127: 1084-1093.

5. Nishihira T, Hashimoto Y, Katayama M, Mori S, Kuroki T (1993) Molecular and cellular features of esophageal cancer cells. Journal of cancer research and clinical oncology 119: 441-449.

6. Fichter CD, Herz C, Munch C, Opitz OG, Werner M, et al. (2011) Occurrence of multipolar mitoses and association with Aurora-A/-B kinases and p53 mutations in aneuploid esophageal carcinoma cells. BMC cell biology 12: 13.

7. Boonstra JJ, van Marion R, Beer DG, Lin L, Chaves P, et al. (2010) Verification and unmasking of widely used human esophageal adenocarcinoma cell lines. Journal of the National Cancer Institute 102: 271-274.

8. Vander Heiden MG, Cantley LC, Thompson CB (2009) Understanding the Warburg effect: the metabolic requirements of cell proliferation. Science 324: 1029-1033.

9. Dang CV (2010) p32 (C1QBP) and cancer cell metabolism: is the Warburg effect a lot of hot air? Molecular and cellular biology 30: 1300-1302.

10. Berridge MV, Herst PM, Tan AS (2010) Metabolic flexibility and cell hierarchy in metastatic cancer. Mitochondrion 10: 584-588.
