# Supplementary material for: Warburg and Crabtree Effects in Premalignant Barrett's Esophagus Cell Lines with Active Mitochondria
Source: PLoS One. 2013 Feb 27;8(2):e56884. doi: 10.1371/journal.pone.0056884 (PMC3584058; doi:10.1371/journal.pone.0056884)
Supplement: Text S2 — Nuclear and mitochondrial genome characterization. This file contains additional details from the nuclear and mitochondrial genome analyses performed. (DOCX) [file pone.0056884.s012.docx]

**Text S2: Nuclear and mitochondrial genome characterization.**

All cell lines were previously characterized by cytogenetic analysis as having genetic instability prior to being adapted to serum-free media ([Palanca-Wessels, Barrett et al. 1998](file:///C:\Users\martin\Desktop\Work\Thesis\Chapter1\for%20DHockenbery\Text%20S2.docx#_ENREF_20)). In this study, we confirmed that serum-adapted CP-B, CP-C and CP-D have the largest number of chromosomal aberrations (Figure S1).

Comparing the cell lines to patient-derived biopsies acquired at several time points, most of the alterations in genomic copy number were consistent between the cell lines and patient biopsies, with the exception of the several notable changes (data not shown). CP-B is clonally related to samples taken from the patient, similar to a clone with deleted 17p, but displays an additional loss of sequence in the 17q chromosomal region and other chromosomal alterations. Losses on chromosome 17 were not observed in most biopsies taken from this patient. Since this patient went on to cancer, this may represent inherent genetic instability in this line. CP-C is nearly identical genetically to biopsies from multiple time points in the patient, with the exception of chromosome 4 and 14. We also observed additional alterations in evolved clones *in vivo*. The differences in genetics observed between biopsies and cell lines may be due to clonal evolution and/or sampling error. Patient biopsies for CP-D were not available for comparison.

The nuclear genome loci of 30 genes of particular importance in regulating glycolysis, oxidative phosphorylation, acid transport and hypoxia resistance were investigated for copy number status (Table S6). CP-A had gain of copy number in three genes, CP-B had single copy deletions in three genes and CP-C had single copy deletions in 11 genes. Calls for CP-D are difficult since by flow cytometry the cell line appears to be a mixture of a "tetraploid" population that also has some alterations with another aneuploid population that has a different subset of alterations. The tetraploid fraction is likely masking deletion events commonly seen in BE samples. In both CP-C and CP-D, hypoxia inducible factor 1-alpha (HIF-1α) has a single copy deletion. With a single HIF-1α copy remaining, the remaining allele may be subject to constitutive upregulation. HIF-1α is negatively regulated post-translationally by a family of oxygen-dependent prolyl and asparagine hydroxylases which modify residues 402, 564 and 803 for ubiquitination by Von-Hippel Lindau ubiquitin ligase which targets the protein for proteosomal degradation [[1](file:///C:\Users\martin\Desktop\Work\Thesis\Chapter1\for%20DHockenbery\Text%20S2.docx#_ENREF_1),[2](file:///C:\Users\martin\Desktop\Work\Thesis\Chapter1\for%20DHockenbery\Text%20S2.docx#_ENREF_2),[3](file:///C:\Users\martin\Desktop\Work\Thesis\Chapter1\for%20DHockenbery\Text%20S2.docx#_ENREF_3),[4](file:///C:\Users\martin\Desktop\Work\Thesis\Chapter1\for%20DHockenbery\Text%20S2.docx#_ENREF_4)]. Modification of any of these residues would constitutively stabilize this transcription factor, causing an upregulation in target glycolysis and hypoxia-resistance genes.

Since the majority of the mitochondrial proteome is coded in the nuclear genome, we analyzed the 1023 nuclear-encoded genes in the four BE cell lines. CP-D had the most nuclear genome alterations which affect mitochondrial genes, however, since this cell line still retains mitochondrial activity these alterations do not appear to produce loss-of-function in the mitochondria (Table S7). Although there are many single-copy mutations in the nuclear-encoded mitochondrial genes, only a single double-deletion was reported: POLRMT, the DNA-directed mitochondrial RNA polymerase, in CP-B. This is the primase required for initiation of DNA synthesis from the light-strand origin of DNA replication. Since this polymerase function is vital to mitochondrial replication, another polymerase must be complementing its function by synthesizing the primer necessary for replication.

Multiple mtDNA mutations were detected in each of the cell lines, ranging from 2 in CP-D to 26 in CP-A (Table S8). The resequencing data from cell line CP-C indicate the presence of multiple sequences, suggesting the presence of multiple mitochondrial genomes in the cell line, representing either a mixed clonal population or mixed mitochondrial species in each cell (heteroplasmy). Having mixed clonal populations in CP-A and CP-C would explain the abundance of mutations found in these cell lines.

1. Epstein AC, Gleadle JM, McNeill LA, Hewitson KS, O'Rourke J, et al. (2001) C. elegans EGL-9 and mammalian homologs define a family of dioxygenases that regulate HIF by prolyl hydroxylation. Cell 107: 43-54.

2. Ivan M, Kondo K, Yang H, Kim W, Valiando J, et al. (2001) HIFalpha targeted for VHL-mediated destruction by proline hydroxylation: implications for O2 sensing. Science 292: 464-468.

3. Jaakkola P, Mole DR, Tian YM, Wilson MI, Gielbert J, et al. (2001) Targeting of HIF-alpha to the von Hippel-Lindau ubiquitylation complex by O2-regulated prolyl hydroxylation. Science 292: 468-472.

4. Hewitson KS, McNeill LA, Riordan MV, Tian YM, Bullock AN, et al. (2002) Hypoxia-inducible factor (HIF) asparagine hydroxylase is identical to factor inhibiting HIF (FIH) and is related to the cupin structural family. The Journal of biological chemistry 277: 26351-26355.
